# Attitudes to kidney donation among primary care patients in rural Crete, Greece

**DOI:** 10.1186/1471-2458-9-54

**Published:** 2009-02-10

**Authors:** Emmanouil K Symvoulakis, Ioannis D Komninos, Nikos Antonakis, Myfanwy Morgan, Athanasios Alegakis, Emmanouil Tsafantakis, Marios Chatziarsenis, Anastas Philalithis, Roger Jones

**Affiliations:** 1 Department of Blood Donation, University General Hospital of Heraklion, Crete, Greece; 2 Vrachasi Practice, General Hospital/Health Centre of Neapolis, Crete, Greece; 3 Primary Health Centre of Anogia, Rethymno, Crete, Greece; 4 Division of Health and Social Care Research, King's College London, London, UK; 5 Biostatistics Laboratory, Faculty of Medicine, University of Crete, Crete, Greece; 6 Department of Social Medicine, Faculty of Medicine, University of Crete, Crete, Greece; 7 Department of General Practice and Primary Care, King's College London, London, UK

## Abstract

**Background:**

In Greece, there is limited research on issues related to organ donation, and the low rate of registration as donors requires explanation. This study reports the findings of a survey of knowledge and attitudes to kidney donation among primary care patients in rural Crete, Greece.

**Methods:**

Two rural primary care settings in the island of Crete, Anogia Health Centre and Vrachasi Practice, were involved in a questionnaire survey. This was conducted among primary care patients (aged 18 years and over) with routine appointments, to assess their knowledge and attitudes to kidney donation. General practitioners (GPs) recruited patients and questionnaires were completed following the patients' medical consultation. Pearson's chi square tests were used and crude odds ratios (OR) with 95% confidence intervals (95% CI) were calculated in order to investigate into the possible associations between the respondents' knowledge, attitudes and specific concerns in relation to their socio-demographic features. Logistic regression analyses were used to examine differences by geographical location.

**Results:**

The 224 (92.5%) of the 242 primary care attenders who were approached agreed to participate. Only 2.2% (5/224) of the respondents carried a donor card. Most participants (84.4%, 189/224) did not feel well informed about registering as a kidney donor. More than half of the respondents (54.3%, 121/223) were unwilling to register as a kidney donor and donate kidneys for transplant after death. Over a third of respondents (35.4%, 79/223) were not confident that medical teams would try as hard as possible to save the life of a person who has agreed to donate organs. People with a higher level of education were more likely to be willing to register as kidney donors [(OR: 3.3; 95% CI: 1.8–6.0), p < 0.001)] and to be less worried about their kidneys being removed after death [(OR: 0.3; 95% CI: 0.1–0.5), p < 0.001)] than those having a lower level of education.

**Conclusion:**

Lack of knowledge and information regarding organ donation and negative attitudes related to registration as donors were the main findings of this study. Efforts should be based on targeting the attitudes to organ donation of individuals and population groups.

## Background

Transplantation of solid organs was considered a promising experimental option only a few decades ago. Advances in understanding the immunologic pathways and improvements in surgical techniques have transformed the hope for success into a reality [1]. Nowdays, transplantation is a common therapeutic strategy for patients with end-stage organ failure [1]. However, increasing demand coupled with the limited number of organs retrieved represents one of the more serious limitations of organ transplantation. Issues related to the physicians' perceptions and more often to the beliefs and the behaviour of potential donors may account for this mismatch [2]. For instance, a lower kidney donation rate occurs among ethnic minorities living in the UK in comparison to the white population [3]. In a recent study, negative attitudes to registration as a donor have been reported among ethnic groups such as black Caribbeans and black Africans living in the UK [4]. In addition, black or Asian people are more frequently affected by chronic conditions such as diabetes and hypertension, conditions that pose a greater risk for end stage kidney disease and increased registration on kidney transplant waiting lists [5]. Changing negative attitudes to registration as a donor involves much more than overcoming one barrier or need for information [4]. Exploring possible variations in willingness to donate among different population sub-groups may help to explain organ donation acceptance through social, cultural or human diversity.

A *Eurobarometer *study, on how Europeans view organ donation and transplantation, carried out in 2006, showed that Greek citizens are less ready to donate their organs after their death or to give consent to an organ being donated from a deceased close family member, than the citizens of Northern Europe [6]. In Greece, the demand for kidney transplants is not satisfied, with a long national waiting list. However the reasons for more negative attitudes to donation in Greece are currently unexplored. This study aims to determine the knowledge, attitudes and concerns shared across the attenders of general practitioners (GPs) in rural primary care in Crete (Greece), establishing possible influential factors or disparities between population sub-groups and allowing a preliminary comparison with data reported elsewhere.

## Methods

### Setting

Two rural primary care settings in the island of Crete, Anogia Health Centre and Vrachasi Practice, were involved in a questionnaire survey. These primary care centres are the main sources of health care delivery in their respective locations, serving a total population of approximately 6000 persons. The local economy of Anogia community is based on farming, agriculture and some rural, mountainous tourism initiatives. Inhabitants of Vrachasi are frequently employed in sea-side tourism-related activities and farming.

Ethics approval was received from the bio-medical committee of General Hospital of Neapolis (Crete, Greece) and permission was obtained from Primary Health Centre of Anogia (Crete, Greece) considering administrative and clinical governance issues related to the regional health care organisation [7]. Funding was not obtained.

### Sampling

Information on organ transplantation at a national level was retrieved, reporting an approximately 0.5% prevalence of donor card carriers in the early 2000s [8]. We hypothesized that the maximum expected prevalence of a positive response on being registered as donors would be 15%, giving a sample size of at least 196 subjects with an error probability of 5% (confidence level 95%) [9]. We aimed to recruit at least 20% more subjects to account for non-participation. Data were collected over an 8 week period between March 2007 and May 2007. One GP in each primary care setting was responsible for carrying out the study and was responsible for recruiting patients. Attenders who had an appointment with the involved GPs for any medical reason were invited to participate, with exclusion criteria being: age under 18 years, emergency care patients, those with chronic diseases leading to severe organ impairment, patients with cognitive and mood disorders, those with a significant audio-visual disorder and verbal expression problems or who were too sick or too elderly to participate. At the end of the consultation, eligible patients were invited to participate in the survey and appropriate explanations provided, including an assurance that the questionnaires were strictly anonymous. After giving informed consent, participants completed the questionnaire without any further interaction with the GP.

### Questionnaire

The questionnaire used for the survey was based on the questionnaire used by Morgan *et al*. in their study on attitudes to kidney donation among ethnic groups in the UK [4]. This comprised 19 questions covering knowledge, attitudes to kidney donation and specific concerns, plus two questions on living donation. Age, sex, education, occupation, religion, nationality and ethnicity were also recorded. Two bilingual researchers translated the questionnaire from English into Greek and a reconciliation process was applied by the two translators and a third bilingual supervisor. A backward translation was performed by an independent translator and the process report including Greek and English versions was sent to the developers in UK. Comprehensibility, reliability and feasibility of the content in the Greek version were checked by testing the questionnaire among 10 respondents.

### Statistical analysis

Data were tabulated and analysed using the SPSS 16.0 statistical package (SPSS Inc., Chigago, IL, USA). In very few cases, not all questionnaire items were fully or clearly completed and they were reported as missing data. Variables were presented as counts and proportions. Pearson's chi square tests were used and crude odds ratios (OR) with 95% confidence intervals (95% CI) were calculated in order to investigate into the possible associations between the respondents' knowledge, attitudes and specific concerns in relation to their socio-demographic features (women vs. men; age < 40 and age 40–59 years vs. age ≥ 60 years; non orthodox vs. orthodox; paid employment vs. non paid employment; higher education vs. lower education and Anogia respondents vs. Vrachasi respondents). In order to further examine the participants responses to each questionnaire item according to their residence (Anogia or Vrachasi location), adjusted OR (with 95% CI) were calculated using multiple logistic regression analyses after adjusting for age, sex, religion, education, and occupational status. *P*-values <*0.05 *were considered as statistically significant.

## Results

Among the 242 patients who were recruited, 224 (92.5%) agreed to participate. Socio-demographic features of the respondents are shown in Table 1. This identifies differences in the age distribution, educational level and occupational status of respondents attending the two centres. Overall 138 (61.6%) of respondents were less than 60 years and the male to female ratio was 0.86 to 1. The respondents were homogeneous in terms of nationality, ethnicity and religion. Two hundred and seven (92.8%) respondents were self-classified as 'Christian Orthodox'. Two hundred twenty and one (99.5%) of the persons who completed the questionnaire were 'White', while two hundred six (92.8%) were Greeks and sixteen (7.2%) were immigrants permanently residing in Greece. One hundred and twelve (50.0%) persons had a paid employment activity and 153 (68.3%) respondents had obtained a secondary or below educational degree.

**Table 1:** Socio-demographic characteristics of the respondents

**Socio-demographics**	**Location**				
	**Vrachasi**		**Anogia**		***P-value***
	n	%	n	%	
**Age (years)**					
< 40	32	25.2	32	33.0	**0.002**
40–59	54	42.5	20	20.6	
≥ 60	41	32.3	45	46.4	
**Sex**					
Male	59	46.5	45	46.4	0.992
Female	68	53.5	52	53.6	
**Religion**					
Orthodox	115	91.3	92	94.8	0.305
Non-Orthodox*	11	8.7	5	5.2	
**Nationality**					
Greek	115	90.6	91	95.8	0.135
Non-Greek**	12	9.4	4	4.2	
**Education**					
Secondary or below	72	56.7	81	83.5	** < 0.001**
Further-commercial/technical	34	26.8	6	6.2	
University/polytechnic	21	16.5	10	10.3	
**Occupation**					
Paid employment	56	44.1	56	57.7	**0.030**
Student	10	7.9	4	4.1	
Not working	18	14.1	4	4.1	
Retired	43	33.9	33	34.1	

* Catholic (n = 5), Muslim (n = 2), Atheist (n = 4), Other (n = 5)

** British (n = 10), Albanian (n = 2), Dutch (n = 1), Russian (n = 1), Bulgarian (n = 1) and Syrian (n = 1)

Table 2 shows that only 2.2% of the respondents were carriers of a donor card. Over 84% of the participants did not feel well informed about registering as a kidney donor. Thirty eight per cent had previously discussed donating kidneys with their partner, family members or friends. More than half of the respondents (54.3%) were unwilling to register as a kidney donor and donate kidneys for transplant after death. Thirty nine per cent were worried about kidneys being removed after death. Over 61% of the respondents were worried that organs might be used without consent for other purposes. About one-quarter of the respondents (25.6%) had concerns that registering to be a donor is like tempting death. Finally, about 15% of the respondents had thoughts that an intact body is needed after death.

**Table 2:** Knowledge, general attitudes and specific concerns of the participants

**Questionnaire's domains**	**NO**	**YES**
	**n (%)**	**n (%)**
**Knowledge**		
Are you registered on the national organ donor register and do you carry a donor card?	219/224 (97.8)	5/224 (2.2)
Did you know it was possible to leave kidneys for transplant after death?	105/223 (47.1)	118/223 (52.9)
Do you feel well informed about registering as a kidney donor?	189/224 (84.4)	35/224 (15.6)
Do you know anyone who has received or is waiting to receive a kidney?	169/224 (75.4)	55/224 (24.6)
**General attitudes**		
Have you ever thought about donating kidneys after death?	135/224 (60.3)	89/224 (39.7)
Would you be willing to register as kidney donor and donate kidneys for transplant after death?	121/223 (54.3)	102/223 (45.7)
Have you ever discussed donating kidneys with partner, family member or friend?	138/224 (61.6)	86/224 (38.4)
Would you be willing to register as a donor if it was not necessary to carry a donor card?	113/224 (50.4)	111/224 (49.6)
Would you oppose a system that made it lawful to take kidneys from an adult who has just died unless that person had forbidden it while he was alive?	148/222 (66.7)	74/222 (33.3)
**Specific concerns**		
If a kidney donor would you mind who received your kidneys after your death?	158/224 (70.5)	66/224 (29.5)
Do you agree that it is important to know that someone else is given a chance of life after donor's death?	28/224 (12.5)	196/224 (87.5)
Are you confident that medical teams will try as hard to save the life of a person who has agreed to donate organs?	79/223 (35.4)	144/223 (64.6)
Are you worried about your kidneys being removed after death?	136/223 (61.0)	87/223 (39.0)
Do you worry that donated organs might be used without consent for other purposes like medical research?	85/223 (38.1)	138/223 (61.9)
Do you find organ donation unacceptable because of religious beliefs?	211/224 (94.2)	13/224 (5.8)
Do you think that registering to be a donor is like tempting death?	166/223 (74.4)	57/223 (25.6)
Do you think that carrying a donor card is like tempting death?	140/223 (62.8)	83/224 (37.2)
Do you agree that donating organs when you die is a good thing to do?	8/219 (3.7)	211/219 (96.3)
Do you think that an intact body is needed after death?	191/224 (85.3)	33/224 (14.7)
Would you consider becoming a live donor if a young child required a kidney?	24/222 (10.8)	198/222 (89.2)
Would you consider becoming a live donor if an adult required a kidney?	64/222 (28.8)	158/222 (71.2)

Totals differ as they exclude missing data

In Table 3 crude odds ratios (OR) and 95% confidence intervals (CI) exploring associations between responses to each questionnaire item and socio-demographic features are shown. Reference categories are also listed. Among the data that achieved statistical significance, women had a significantly lower OR of feeling well informed about registering as a kidney donor [(OR: 0.5; 95% CI: 0.2–1.0), *p *< 0.05)] when compared with men but were less likely to think that registering to be a donor is like tempting death [(OR: 0.4; 95% CI: 0.2–0.8), *p *< 0.01)] than men. Respondents aged under 40 years were more likely to be willing to register as a kidney donor and donate kidneys for transplant [(OR: 3.7; 95% CI: 1.9–7.5), *p *< 0.001)] than respondents aged 60 years or over. Respondents aged between 40 and 59 years were also more likely to be willing to register as a kidney donor and donate kidneys for transplant [(OR: 4.4; 95% CI: 2.2–8.7), *p *< 0.001)] than respondents aged 60 years or over. Attenders who were not Greek Orthodox had higher OR of feeling well informed [(OR: 6.7; 95% CI: 2.3–19.2), *p *< 0.001)] when compared with the Orthodox group. Respondents in paid employment were more likely to have previously discussed donating kidneys with a partner, family members or friends [(OR: 2.5; 95% CI: 1.4–4.4), *p *< 0.001)] than those not having a paid activity (students, the unemployed and retired). People with a higher education level (commercial/technical and university/polytechnic) had a higher OR of being registered as donor [(OR: 9.1; 95% CI: 1.0–82.8), *p < 0.05*)] than those having a lower level of education (secondary or below). Respondents of this group were more likely to know that it was possible to leave kidneys for transplant [(OR: 3.8; 95% CI: 2.0–7.1), *p *< 0.001)], to be willing to register as kidney donors [(OR: 3.3; 95% CI: 1.8–6.0), *p *< 0.001)], to be less worried about kidneys being removed after death [(OR: 0.3; 95% CI: 0.1–0.5), *p *< 0.001)] than those having a lower level of education (secondary or below). People with higher education were less likely to think that carrying a donor card is like tempting death [(OR: 0.3; 95% CI: 0.2–0.6), *p *< 0.001)] than those having a lower level of education.

**Table 3:** Knowledge, attitudes and specific concerns in relation to the socio-demographic features. Crude Odds Ratios (95%CI).

**Conceptual Domains**	**Sex^1^**	**Age^2^**		**Religion^3^**	**Occupation^4^**	**Education^5^**	**Location^6^**
	Women	< 40 years	40–59 years	Non-Orthodox	Paid employment	Higher than secondary	Anogia respondents
Registered on the national organ donor register and carry donor card	0.6 (0.1–3.5)	*	*	3.4 (0.4–32.2)	4.1 (0.5–37.3)	9.1 (1.0–82.8)^*a*^	0.3 (0–2.9)

Know that it was possible to leave kidneys for transplant	0.9 (0.5–1.5)	2.4 (1.2–4.7)^*b*^	2.1 (1.1–3.9)^*a*^	1.4 (0.5–4.0)	1.0 (0.6–1.7)	3.8 (2.0–7.1)^*c*^	0.2 (0.1–0.3)^*c*^

Feel well informed about registering as a kidney donor	0.5 (0.2–1.0)^*a*^	2.2 (0.9–5.5)	1.8 (0.7–4.6)	6.7 (2.3–19.2)^*c*^	1.6 (0.8–3.4)	1.3 (0.6–2.8)	2.2 (1.1–4.7)

Know someone who has received or is waiting to receive a kidney	1.1 (0.6–1.9)	1.2 (0.6–2.7)	1.8 (0.9–3.8)	1.4 (0.5–4.3)	1.9 (1.0–3.5)^*a*^	1.6 (0.9–3.1)	1.8 (1.0–3.4)

Have thought about donating kidneys after death	1.0 (0.6–1.8)	2.8 (1.4–5.5)^*b*^	1.4 (0.7–2.7)	0.9 (0.3–2.6)	1.2 (0.7–2.1)	1.9 (1.1–3.4)^*a*^	1.7 (1.0–3.0)^*a*^

Would be willing to register as kidney donor and donate kidneys for transplant	1.2 (0.7–2.0)	3.7 (1.9–7.5)^*c*^	4.4 (2.2–8.7)^*c*^	1.6 (0.6–4.4)	1.8 (1.1–3.1)^*a*^	3.3 (1.8–6.0)^*c*^	0.5 (0.3–0.8)^*b*^

Have discussed donating kidneys with partner, family member or friend	0.6 (0.3–1.0)	1.9 (1.0–3.8)	1.4 (0.1–2.7)	3.9 (1.3–11.6)^*b*^	2.5 (1.4–4.4)^*c*^	2.5 (1.4–4.5)^*b*^	2.1 (1.2–3.7)^*b*^

Would be willing to register as a donor if it was not necessary to carry a donor card	0.9 (0.5–1.5)	2.3 (1.2–4.5)^*a*^	1.9 (1.0–3.6)^*a*^	1.3 (0.5–3.7)	1.0 (0.6–1.8)	2.7 (1.5–4.9)^*c*^	0.4 (0.3–0.8)^*b*^

Would oppose system that made it lawful to take kidneys from an adult who has just died unless that person had forbidden it while he was alive	0.8 (0.4–1.3)	1.3 (0.7–2.7)	1.5 (0.8–3.0)	1.2 (0.4–3.5)	1.8 (1.0–3.1)^*a*^	0.9 (0.5–1.6)	0.8 (0.5–1.5)

If a kidney donor would mind who received kidneys after death	1.1 (0.6–1.9)	0.9 (0.4–1.8)	0.6 (0.3–1.2)	0.5 (0.1–1.9)	0.8 (0.4–1.4)	0.7 (0.4–1.3)	1.1 (0.6–2.0)

Agree that it is important to know that someone else is given a chance of life	1.0 (0.5–2.2)	1.4 (0.4–4.3)	0.5 (0.2–1.2)	0.2 (0.1–0.6)^*b*^	1.2 (0.5–2.6)	1.0 (0.4–2.3)	4.1 (1.5–11.1)^*b*^

Are confident that medical teams will try as hard to save the life of a person who has agreed to donate organs	1.0 (0.6–1.8)	1.2 (0.6–2.6)	0.7 (0.4–1.3)	0.7 (0.2–1.9)	1.3 (0.7–2.3)	1.0 (0.6–1.8)	3.6 (2.0–6.7)^*c*^

Are worried about kidneys being removed after death	0.6 (0.4–1.1)	0.7 (0.4–1.4)	0.6 (0.3–1.1)	0.5 (0.2–1.6)	1.1 (0.6–1.8)	0.3 (0.1–0.5)^*c*^	3.8 (2.2–6.8)^*c*^

Worry that donated organs might be used without consent for other purposes like medical research	0.9 (0.5–1.5)	1.0 (0.5–1.9)	0.8 (0.4–1.5)	2.9 (0.8–10.4)	0.9 (0.5–1.5)	1.3 (0.7–2.4)	0.4 (0.2–0.7)^*c*^

Find organ donation unacceptable because of religious beliefs	1.0 (0.3–3.1)	0.2 (0.02–1.3)	0.6 (0.2–1.9)	4.5 (1.1–18.6)^*a*^	0.8 (0.3–2.6)	0.2 (0.02–1.3)	1.1 (0.4–3.5)

Think that registering to be a donor is like tempting death	0.4 (0.2–0.8)^*b*^	0.5 (0.3–1.1)	0.4 (0.2–0.8)^*a*^	0.4 (0.1–1.8)	0.7 (0.4–1.3)	0.4 (0.2–0.8)^*b*^	2.7 (1.5–5.1)^*c*^

Think that carrying a donor card is like tempting death	0.5 (0.3–0.9)^*a*^	0.7 (0.3–1.3)	0.4 (0.2–0.8)^*a*^	0.5 (0.2–1.7)	1.1 (0.6–1.8)	0.3 (0.2–0.6)^*c*^	4.7 (2.6–8.5)^*c*^

Agree that donating organs when you die is a good thing to do	1.2 (0.3–4.8)	*	0.8 (0.2–3.5)	0.1 (0.02–0.5)^*c*^	1.0 (0.2–4.1)	3.4 (0.4–28.2)	0.5 (0.1–2.0)

Think that an intact body is needed after death	0.8 (0.4–1.6)	0.5 (0.2–1.2)	0.5 (0.2–1.1)	2.0 (0.6–6.8)	0.9 (0.4–2.0)	0.2 (0.1–0.6)^*b*^	2.6 (1.2–5.7)^*a*^

Would consider becoming a live donor if a young child required a kidney	1.0 (0.4–2.3)	1.0 (0.4–2.7)	2.1 (0.7–6.2)	0.5 (0.1–1.9)	1.0 (0.4–2.3)	1.5 (0.6–3.9)	1.1 (0.5–2.5)

Would consider becoming a live donor if an adult required a kidney	1.3 (0.7–2.4)	1.1 (0.5–2.2)	0.9 (0.4–1.7)	0.9 (0.3–2.7)	1.2 (0.7–2.1)	0.9 (0.5–1.6)	4.3 (2.2–8.6)^*c*^

* Not determined

^1^Reference category: Men

^2^Reference category: ≥ 60 years

^3^Reference category: Orthodox

^4^Reference category: Those not having a paid employment (students, not working, retired)

^5^Reference category: Those having a secondary education level or below

^6^Reference category: Vrachasi respondents

a: p < 0.05

b: p < 0.01

c: p < 0.001

Table 4 shows the adjusted OR (95% CI) estimates of each questionnaire item with location (Anogia Health Centre vs. Vrachasi Practice) after adjusting for age, sex, religion, education, and occupation. Among the data that met statistical significance, respondents from Anogia primary care setting were less likely to know that it was possible to leave kidneys for transplant [(OR: 0.2; 95% CI: 0.1–0.4), *p *< 0.001)], had higher OR of being confident that medical teams will try hard to save the life of a person who has agreed to donate organs [(OR: 3.6; 95% CI: 1.8–7.1), *p *< 0.001)] and to feel less worried that donated organs might be used without consent for other purposes such as medical research [(OR: 0.4; 95% CI: 0.2–0.7), *p *< 0.001)] than those of Vrachasi primary care setting. However, respondents of Anogia were more likely to be generally worried about kidneys being removed after death [(OR: 3.2; 95% CI: 1.7–6.0), *p *< 0.001)], and to think that carrying a donor card is like tempting death [(OR: 3.8; 95% CI: 2.0–7.2), *p *< 0.001)] than those of Vrachasi. Finally, inhabitants of Anogia were more likely to consider becoming a living donor if an adult required a kidney [(OR: 5.3; 95% CI: 2.5–11.3), *p *< 0.001)] than inhabitants of Vrachasi.

**Table 4:** Adjusted Odds Ratios (95% CI) estimates for location (adjusting for age, sex, religion, education, and occupation)

**Conceptual Domains**	**Positive responses**	**Vrachasi** **respondents** (n = 127)	**Anogia** **respondents** (n = 97)	**Adjusted** **OR (95%CI)**	**P-value**
Registered on the national organ donor register and carry donor card	5	4 (3.1%)	1 (1.0%)	0.8 (0.1–9.0)	0.852
Know that it was possible to leave kidneys for transplant	118	89 (70.6%)	29 (29.9%)	0.2 (0.1–0.4)	** < 0.001**
Feel well informed about registering as a kidney donor	35	14 (11.0%)	21 (21.6%)	3.2 (1.3–8.0)	**0.013**
Know someone who has received or is waiting to receive a kidney	55	25 (19.7%)	30 (30.9%)	2.4 (1.2–5.0)	**0.016**
Have thought about donating kidneys after death	89	43 (33.9%)	46 (47.4%)	2.5 (1.3–4.7)	**0.005**
Would be willing to register as kidney donor and donate kidneys for transplant	102	68 (54.0%)	34 (35.1%)	0.6 (0.3–1.1)	0.093
Have discussed donating kidneys with partner, family member or friend	86	39 (30.7%)	47 (48.5%)	2.9 (1.5–5.8)	**0.002**
Would be willing to register as a donor if it was not necessary to carry a donor card	111	74 (58.3%)	37 (38.1%)	0.5 (0.3–1.0)	**0.041**
Would oppose system that made it lawful to take kidneys from an adult who has just died unless that person had forbidden it while he was alive	74	44 (34.9%)	30 (31.2%)	0.7 (0.4–1.3)	0.250
If a kidney donor would mind who received kidneys after death	66	36 (28.3%)	30 (30.9%)	1.0 (0.5–1.8)	0.885
Agree that it is important to know that someone else is given a chance of life after donor's death	196	104 (81.9%)	92 (94.8%)	4.3 (1.4–13.3)	**0.010**
Are confident that medical teams will try as hard to save the life of a person who has agreed to donate organs	144	67 (52.8%)	77 (80.2%)	3.6 (1.8–7.1)	** < 0.001**
Are worried about kidneys being removed after death	87	32 (25.4%)	55 (56.7%)	3.2 (1.7–6.0)	** < 0.001**
Worry that donated organs might be used without consent for other purposes like medical research	138	90 (70.9%)	48 (50.0%)	0.4 (0.2–0.7)	** < 0.001**
Find organ donation unacceptable because of religious beliefs	13	7 (5.5%)	6 (6.2%)	0.6 (0.2–2.4)	0.494
Think that registering to be a donor is like tempting death	57	22 (17.3%)	35 (36.5%)	2.4 (1.2–4.9)	**0.016**
Think that carrying a donor card is like tempting death	83	28 (22.0%)	55 (57.3%)	3.8 (2.0–7.2)	** < 0.001**
Agree that donating organs when you die is a good thing to do	211	119 (97.5%)	92 (94.8%)	0.6 (0.1–3.9)	0.561
Think that an intact body is needed after death	33	12 (9.4%)	21 (21.6%)	1.8 (0.8–4.3)	0.184
Would consider becoming a live donor if a young child required a kidney	198	112 (88.9%)	86 (89.6%)	1.6 (0.6–4.2)	0.357
Would consider becoming a live donor if an adult required a kidney	158	75 (59.5%)	83 (86.5%)	5.3 (2.5–11.3)	** < 0.001**

## Discussion

The identification of specific areas of knowledge, attitudes and perceptions related to kidney donation helps to achieve a better understanding of variations in willingness to donate. A striking contrast was found in our study between people that were actually registered as donors and those who had stated that they were willing to register as kidney donors (Table 2). Only a minority of respondents felt well informed about registering as a kidney donor. Most respondents were concerned that donated organs might be used without consent for other purposes.

Although the questionnaire has been used previously [4], in some cases the wording may be considered simplified (e.g. "donation after death" does not distinguish between heart beating and non-heart beating organ donation). On the other hand, this may be appropriate in view of the complexity of the issues. Some important findings of our study were related to the likely positive impact of education on certain attitudes to kidney organ donation. Conesa *et al.*, suggest that teenagers with a higher education have more favourable attitudes towards organ donation and that those who have left school early have a more negative approach [10]. Confirming the importance of education, we suggest that efforts should be focused on groups with lower level of education by introducing and discussing issues including organ donation and end of life aspects in the community.

The apparent contradiction between the positive responses to registering as a donor, with or without a donor card (45.7% and 49.6%) and accepting presumed consent (66.7%) may be explained by the fact that the latter does not involve any specific action by the respondent. We also found that people in paid employment were more likely to be willing to register as kidney donors and donate kidneys for transplant when compared with those not having a paid activity. At the same time, they were more likely to oppose a system that made it lawful to take kidneys from an adult who has just died unless that person had forbidden it while he was alive. It appears that people, through their occupational activity, are probably prone to interactions towards more positive personal views on specific areas of organ donation but are also concerned about the integrity of consent and donation procedures.

The Greek Orthodox Church does not oppose organ donation [11]. Our finding that most respondents (94.2%) do not consider organ donation unacceptable because of religious beliefs was not unexpected. From our observations, sometimes Greek Orthodox people have a "natural tendency" to explain events or shape attitudes emotionally. They may be ready to state unanimously that donating organs after death is a good thing to do but only half of them declare their willingness to register as kidney donors. Non-Orthodox respondents were more likely to feel well informed about registering as kidney donors and to have previously discussed donating kidneys with partner, family members or friends compared to Orthodox respondents but were less likely to agree that it is important to know that someone else is given a chance of life, to agree that donating organs when you die is a good thing to do and were more likely to find organ donation unacceptable because of religious beliefs. The limited size of the non-Orthodox group, and its mixed composition, may explain some heterogeneity in responses within this group. To avoid similar problems in interpretation we did not include nationality among the socio-demographic variables in Table 3.

Adjusted OR (95% CI) estimates for location (Anogia vs. Vrachasi), after adjusting with age, sex, religion, education, and occupation revealed a different pattern of responses (Table 4). Patients at Anogia were more consistent in their views on certain topics, such as reporting high levels of information on registering as donors, prior involvement in thinking about or discussing donation issues, high level of confidence in medical teams and limited awareness about the use of transplants than facility users of Vrachasi. On the other hand respondents from Anogia were more likely to worry about kidneys being removed after death and to think that registering or carrying a donor card is like tempting death when compared with those from Vrachasi. Respondents from Anogia had lower OR of knowing that it was possible to leave kidneys for transplant, in contrast with the fact that they said they felt well informed about registering as kidney donors.

Certain attitudes among the inhabitants of Anogia may be related to "stricter" patterns of traditional family and community life. What is morally appropriate, socially desiderable and personally acceptable becomes complex in small communities or subgroups with social and cultural idiosyncrasies preserved for decades such as in Anogia. Influential factors linked to social, economic, cultural features [12,13] may have a significant impact on shaping the conceptual approach of a population group to organ donation. Evaluation of attitudes using standardized assessment procedures is essential in order to ensure unbiased measurements, but may not detect more subtle variations in personal beliefs that may affect the willingness to donate [14]. For example, fear of the afterlife has a strong effect on human perceptions and beliefs. The responses of the Vrachasi patients, such as thoughts that registering or carrying a donor card is like tempting death, may reflect a different way of personal or social "conscience development", related to more pragmatic approaches to life. Qualitative research methods would be a more appropriate way of exploring this complex area.

In Greece, it is possible to donate organs or tissues to a close relative while you are still alive. When participants were asked if they would consider becoming living donors in the case of a young child or an adult requiring a kidney, most of them declared their willingness to donate. Response content differs due to the type of donation, living or cadaveric. In a study on determinants of willingness to donate living related and cadaveric organs, it was shown that ethnic or socio-economic features, which explained the greatest amount of variation in willingness to participate in cadaveric donation, were not related to willingness to become a living donor [14]. It is worth noting that patients at Anogia had higher OR of considering becoming a living donor if an adult required a kidney compared to those of Vrachasi. Living donation may acquire a different 'emotional' content within a broader family [15] or immediate community. Responses to the related topic might be influenced by what is important for the integrity of a community or desiderable for a group in terms of 'social coherence'. Additionally, living donation is a hypothetical scenario that, at the time of responding to a survey, does not require an immediate decision which may affect one's health. Both groups had a similar approach to the case of a young child requiring a kidney and their responses were not found to be significantly different.

The limitations of our study deserve some comments. The questionnaires were completed in a period of few weeks. We can not exclude possible interactions among the members of small communities and/or predict how these interactions might influence the response content. Asking to complete a questionnaire in a primary care setting, after consultation, may decrease refusals but at the same moment, may enhance deductive reasoning of what is expected as a response in a field with social content dimensions. Our study was based in rural settings, with a need for comparable information based on similar studies in urban settings. The results of this study, carried out in a specific population, are not generalisable to the whole of Greece. However, our findings are comparable to the responses of Greek citizens to the *Special Eurobarometer *survey, when similar topics as willingness to donate after death or involvement of family in a discussion on donation were examined [6].

## Conclusion

Views on kidney donation appear to be strongly related to interactions between limited knowledge, lack of information and pre-existent neutral or negative beliefs about being a donor, together with a more subtle influence of 'spiritual' concerns or perceptions. It seems that the complexity of human nature, socio-cultural influences and the interplay between personal and social conscience [10,16] represent important determinants on shaping beliefs and general opinions on kidney donation. Our study provides the base-line information for comparisons and future monitoring. Research needs to be carried out in order to collect further information on possible variations occurring between rural and urban settings to develop a better understanding of socio-demographic diversity in shaping attitudes to donation. Efforts should be based on targeting the remodeling of persons' conscience as individuals and as groups. Variables related to group identity and belonging may explain variations of willingness to donate beyond 'stereotypes' and 'narrow descriptions' [17]. Health educational strategies should be targeted at personal, family, and community levels. The role of primary health care may be crucial [18]. Developing and testing strategies through the use of primary care settings as multi-level information vectors may help to deal with people's uncertainties and their confidence and trust in the medical system. Coordinated initiatives including information, education campaigns and knowledge dissemination are of great importance for opening a modern public debate on end of life issues. Distorted beliefs, negative or ambivalent attitudes, indifference and lack of knowledge and trust in health care systems often are more harmful than chronic diseases and potentially cost lives. Policy developers and health care providers should focus on how this situation can be reversed for the purpose of increasing donation consent rates in the future.

## Competing interests

The authors declare that they have no competing interests.

## Authors' contributions

RJ, EKS, MM were involved with the study conception. EKS prepared the manuscript. IDK and NA recruited the patients. IDK, NA, ET and MC were involved in organising and performing the data acquisition. AP provided information on intellectual and technical issues of the project. AA carried out the statistical analysis. AP, RJ, MM and EKS were involved in revising the article for important intellectual content and editing details. All authors have read and approved the manuscript.

## Pre-publication history

The pre-publication history for this paper can be accessed here:


